# Social Sciences section of The Journals of Gerontology Series B: Featured 2024 Editor’s Choice Articles

**DOI:** 10.1093/geroni/igaf122.422

**Published:** 2025-12-31

**Authors:** Jessica Kelley

## Abstract

Social science inquiry on age, aging, and the life course spans many topics and methodologies. This symposium highlights papers that were selected as Editor’s Choice articles in the Social Sciences section of The Journals of Gerontology, Series B: Psychological Sciences and Social Sciences in 2024. These papers highlight methodological innovations, important advancements in our state of knowledge in an area, or emerging issues in the study of aging and older adults. Namkung and Sung apply an age-period-cohort analysis to tracking chronic disease prevalence among older Koreans. Reyes, employs narrative qualitative methods to explore how older African American and Latine immigrants define their civic role and relationship to their new country. Thomeer et al. employ life history data to characterize how childbearing histories impact midlife cognition. Sheftel et al. examine how transitions into loneliness following a range of specific life events may be similar or different across twenty countries. Finally, Brady et al. incorporate features of one’s current work experience in impacting mental health when facing parental caregiving.

